# Depression as a prognostic factor for conversion from mild cognitive impairment to all-cause dementia and Alzheimer's disease: a systematic review and meta-analysis of longitudinal studies

**DOI:** 10.3389/fpubh.2026.1831762

**Published:** 2026-05-21

**Authors:** Madina Kassymzhanova, Kuanysh Shonbay, Olga M. Shikhova, Natalya Raspopova, Indira Karibayeva

**Affiliations:** 1 Department of Psychiatry and Narcology, Asfendiyarov Kazakh National Medical University, Almaty, Kazakhstan; 2 Higher School of Psychology, Turan University, Almaty, Kazakhstan; 3 Department of Psychiatry and Narcology, Kazakh-Russian Medical University, Almaty, Kazakhstan; 4 Department of Health Policy and Community Health, Jiann-Ping Hsu College of Public Health, Georgia Southern University, Statesboro, GA, United States; 5 Department of Research Management, JSC Research Institute of Cardiology and Internal Diseases, Almaty, Kazakhstan

**Keywords:** all-cause dementia, Alzheimer's disease (AD), cognitive decline, depression, mild cognitive impairment, neuropsychiatric symptoms, prognostic factors, systematic review

## Abstract

**Background:**

Mild cognitive impairment (MCI) is a transitional stage between normal aging and dementia, with annual conversion rates to Alzheimer's disease (AD) or all-cause dementia estimated at 10%−15%. The role of depression as a prognostic factor for dementia progression remains unclear. This systematic review and meta-analysis aimed to clarify the association between depression and risk of conversion from MCI to AD and all-cause dementia.

**Methods:**

We conducted a systematic review and meta-analysis of longitudinal cohort studies following PRISMA 2020 guidelines. PubMed, PsycINFO, Web of Science, and Scopus were searched. Eligible studies included adults with MCI, depression assessed by clinical diagnosis or validated scales, and reported hazard ratios (HRs) for progression to dementia. Random-effects meta-analyses were performed for unadjusted and adjusted HRs. Risk of bias was assessed using the Newcastle–Ottawa Scale, and certainty of evidence was evaluated with GRADE.

**Results:**

Seventeen studies were included, with follow-up ranging from 6 months to 12 years. In unadjusted analyses, baseline depression was associated with increased risk of progression overall (HR 1.66, 95% CI 1.22–2.26); however, heterogeneity across studies was high (*I*^2^ = 99.1%), limiting confidence in the pooled estimate. The association was significant for progression to AD (HR 1.57, 95% CI 1.15–2.15*; I*^2^ = 99.4%), but not for all-cause dementia (HR 1.95, 95% CI 0.85–4.48; *I*^2^ = 90%). In adjusted analyses, depression remained associated with increased progression risk overall (HR 1.21, 95% CI 1.05–1.39), with high heterogeneity (*I*^2^ = 99.8%). The association was statistically significant for all-cause dementia (HR 1.24, 95% CI 1.01–1.52; *I*^2^ = 78.8%), but not for AD (HR 1.18, 95% CI 0.95–1.47; *I*^2^ = 99.8%). Sensitivity analyses confirmed robustness of findings, and publication bias was not detected. Certainty of evidence was rated very low due to heterogeneity.

**Conclusion:**

Depression appears to be associated with an increased risk of progression from MCI to dementia; however, the very high heterogeneity and very low certainty of evidence substantially limit confidence in the magnitude and consistency of this association. These findings highlight the importance of depression screening and management in MCI populations. Integrating mental health care into cognitive disorder clinics may improve patient outcomes and potentially delay dementia onset.

**Systematic Review registration:**

identifier: CRD420261308245.

## 1 Introduction

According to a United Nations report on aging, by the 2070s the number of people aged 65 and older is expected to reach 2.2 billion, exceeding the global population of children under 18 (1). By the mid-2030s, those aged 80 and above (about 265 million) will outnumber infants (1). This global demographic transition toward an aging population has led to a sharp rise in age-related cognitive disorders. According to the Global Burden of Disease Study, among individuals aged ≥65 years, the disability-adjusted life years (DALYs) rate attributable to dementia was approximately 404.19 per 100,000 population in 2021 (2). Globally, dementia also ranks fourth among the leading contributors to nervous system DALYs (3).

Mild cognitive impairment (MCI) is increasingly recognized as a critical stage between normal cognitive aging and dementia (4). Although individuals with MCI maintain relative independence in daily functioning, they exhibit measurable cognitive decline that places them at heightened risk for progression to dementia (5). Therefore, MCI is increasingly viewed as a key focus for therapeutic intervention and proper management (6).

Longitudinal studies have consistently demonstrated that MCI is a heterogeneous condition, with annual conversion rates to dementia ranging from 6% to 15% (7). Among these, Alzheimer's disease (AD) is the most common outcome, although progression to all-cause dementia is also frequent. Meta-analytic evidence suggests that nearly 25% of individuals with MCI progress to AD over a 3–6-year period (8). This variability in progression underscores the importance of identifying prognostic factors that may accelerate or mitigate the risk of conversion (9). Such knowledge can inform targeted interventions, reduce disease burden, and improve quality of life for affected populations.

Depression is one of the most common neuropsychiatric symptoms observed in individuals with MCI. Importantly, longitudinal evidence indicates that nearly one third of patients with MCI eventually progress to dementia (10). Previous studies have suggested that depression may accelerate cognitive decline and increase the risk of progression to dementia, potentially through mechanisms involving neuroinflammation, hippocampal atrophy, or vascular changes (11–13). However, findings across studies remain inconsistent, with some reporting strong associations and others finding no significant effect.

To address this gap, the present study conducted a systematic review and meta-analysis of longitudinal cohort studies examining the association between depression and risk of progression from MCI to AD and all-cause dementia. By pooling hazard ratios across diverse populations and settings, this study aimed to provide a comprehensive and evidence-based assessment of whether depression serves as a prognostic factor for conversion, thereby informing clinical practice and guiding future research.

## 2 Methods

### 2.1 Ethical declaration and study registration

This systematic review and meta-analysis was conducted in accordance with the study protocol approved by the Institutional Review Board of Asfendiyarov Kazakh National Medical University (protocol number 752; approval date: 29 May 2019). Because this work represents a secondary analysis of previously published data, the Board did not require an updated approval. The review protocol was prospectively registered in PROSPERO (CRD420261308245) and the methods were developed a priori. The review was conducted and reported in line with the Preferred Reporting Items for Systematic Reviews and Meta-Analyses 2020 (PRISMA 2020) statement (14). All stages of the review—including development of eligibility criteria, search strategy, data extraction template, study selection, critical appraisal, and data extraction—were performed independently by two reviewers (M.K. and K.Sh.). Discrepancies were reconciled through discussion and adjudication by a third reviewer (N.R.).

### 2.2 Eligibility criteria

Eligibility criteria were defined using the Patient (P), Exposure (E), Comparator (C), Outcome (O), Study design (S) framework, aligned with the registered protocol. Population: We included adults diagnosed with MCI using any of the recognized diagnostic criteria. We excluded studies of cognitively normal adults who progressed to MCI or dementia. Exposure: We included depression defined by clinical diagnosis/records or validated depression scales. Studies assessing other neuropsychiatric symptoms (e.g., anxiety, apathy, sleep disturbance) without a depression exposure were excluded. Comparator: No explicit comparator was required because the review addressed depression as a prognostic factor; eligible studies reported hazard ratios comparing participants with vs. without depression, or per-unit increases in depression measures. Outcomes: The main outcomes were conversion from MCI to (1) all-cause dementia and (2) AD, quantified as hazard ratios (HRs). Study design and context: We included prospective or retrospective longitudinal cohort studies conducted in clinical or community settings with a minimum follow-up duration of at least 6 months. We excluded interventional studies, randomized controlled trials, reviews, editorials, and commentaries. Only English-language, published studies were eligible, with no restrictions on publication year.

### 2.3 Search strategy

The search strategy was developed after a preliminary search in PubMed, which specified PubMed, PsycINFO, Web of Science and Scopus as core databases. Searches were limited to human studies published in English and were not restricted by year of publication. To ensure comprehensive coverage and capture the full breadth of the literature, the search strategy intentionally included a wide range of neuropsychiatric terms (e.g., anxiety, apathy, and related symptoms) alongside depression-related keywords. This approach was designed to maximize sensitivity and minimize the risk of missing relevant studies. However, during the screening process, the focus was restricted specifically to depression as the exposure of interest in accordance with the predefined eligibility criteria. Example PubMed search strategy (executed in December, 2025; filters: English; humans; no year limits): (“Mild Cognitive Impairment”[MeSH] OR “Mild Cognitive Impairment”[tiab] OR MCI[tiab]) AND (“Depression”[MeSH] OR “Depressive Disorder”[MeSH] OR “depressive symptoms”[tiab] OR depression[tiab] OR anxiety[tiab] OR “Anxiety Disorders”[MeSH] OR apathy[tiab] OR “neuropsychiatric symptoms”[tiab] OR “neurobehavioral symptoms”[tiab]) AND (“Alzheimer Disease”[MeSH] OR “Dementia”[MeSH] OR “Alzheimer's disease”[tiab] OR dementia[tiab] OR conversion[tiab] OR progression[tiab] OR transition[tiab]) AND (longitudinal[tiab] OR cohort[tiab] OR “prospective study”[tiab] OR “follow-up”[tiab]) NOT (“Randomized Controlled Trial”[Publication Type] OR “randomized controlled trial”[tiab] OR “Review”[Publication Type] OR review[tiab]).

### 2.4 Study selection and data extraction

Records were deduplicated and screened in two stages: (1) title/abstract screening and (2) full-text eligibility assessment. Both stages were conducted independently by two reviewers (M.K. and K.Sh.), with discrepancies resolved by consensus and third-reviewer adjudication (N.R.). This process followed the protocol's requirement for independent screening and a predefined discrepancy-resolution approach. A standardized extraction table was developed prior to data collection. Two reviewers independently extracted study characteristics (country, setting, sample size, name of the study, follow-up duration, MCI diagnostic criteria, outcome ascertainment), exposure definitions (depression assessment method), adjustment sets, and effect estimates (HRs with 95% confidence intervals). The protocol specified that authors would be contacted when necessary data were missing or unclear. Any disagreements were resolved through discussion and third-reviewer adjudication (N.R.).

### 2.5 Risk of bias assessment

Risk of bias was assessed independently by two reviewers (M.K. and K.Sh.) using the Newcastle–Ottawa Scale (NOS) for cohort studies, with disagreements resolved by discussion and adjudication by a third reviewer (N.R.). The NOS evaluates methodological quality across three domains: Selection (4 items), Comparability (1 item, awarding up to 2 stars), and Outcome (3 items), for a maximum total of 9 stars. Each study was scored by awarding stars for items judged to have low risk of bias, and an overall NOS score (0–9) was calculated by summing stars across domains. Consistent with common NOS interpretation in cohort reviews, overall study quality was categorized as high (7–9 stars), moderate (4–6 stars), or low (0–3 stars). Domain-level judgments (selection, comparability, and outcome) were also retained to support transparency in study appraisal and to inform certainty-of-evidence judgments.

### 2.6 Certainty of evidence (GRADE)

The certainty of evidence for each meta-analytic outcome was assessed using the GRADE framework (15, 16). Because the evidence base comprised observational cohort studies, the starting certainty was low, and certainty could be rated down based on: (1) risk of bias (NOS findings), (2) inconsistency (unexplained heterogeneity across studies), (3) indirectness (differences in population, exposure definition, outcome ascertainment, or setting relative to the review question), (4) imprecision (wide confidence intervals and/or limited number of events/studies), and (5) publication bias (funnel plot asymmetry and Egger's test when applicable). Certainty could be rated up when appropriate based on (1) large magnitude of effect, (2) dose–response gradient, and/or (3) residual confounding likely minimizing the observed association. Final certainty ratings were assigned as high, moderate, low, or very low for each pooled outcome, and judgments were documented with explicit rationale (17).

### 2.7 Meta-analysis plan

HR were used as the common effect measure to quantify the association between depression in adults with MCI and risk of progression to all-cause dementia and AD. HRs were log-transformed prior to pooling, and standard errors were derived from reported 95% confidence intervals when required. Separate random-effects meta-analyses were conducted for each outcome using the restricted maximum likelihood (REML) estimator to account for between-study heterogeneity. Primary analyses pooled unadjusted and most fully adjusted HRs separately to distinguish crude associations from estimates that better account for confounding. Heterogeneity was evaluated using Cochran's Q, *I*^2^, and τ^2^. Subgroup analyses were conducted by progression outcome (MCI-to-AD vs. MCI-to-all-cause dementia). Meta-regression by publication year and leave-one-out analyses were performed to explore potential sources of heterogeneity. Publication bias was assessed using funnel plots and Egger's regression test when at least 10 studies were available. All steps of the meta-analysis were conducted using R version 4.5.1 (2025-06-13) (18) within the RStudio environment (version 2025.9.0.387) (19).

## 3 Results

### 3.1 Description of the included studies

**Figure 1:**
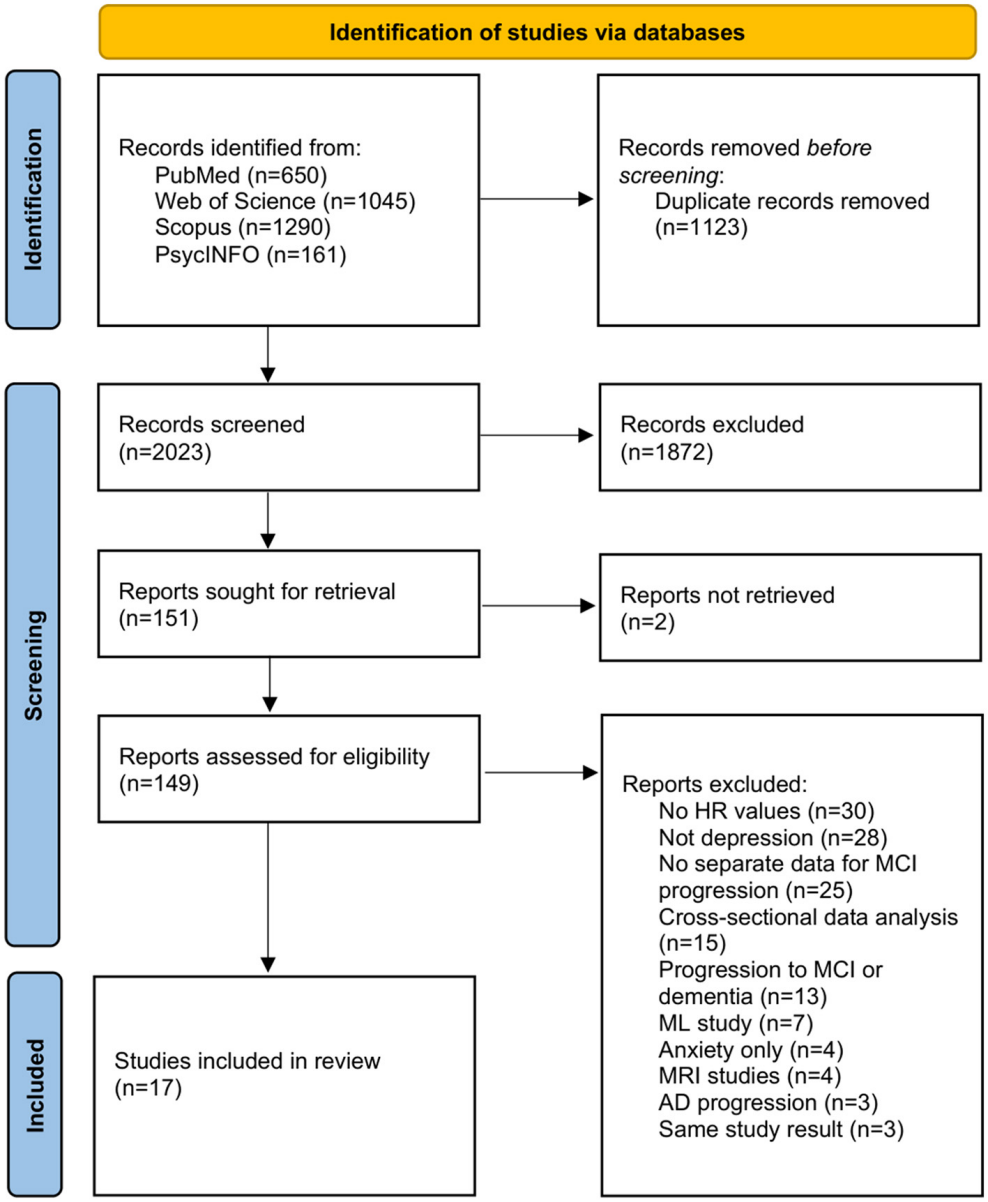
PRISMA flowchart of study selection and inclusion.

Figure 1 presents the PRISMA flowchart for study identification, screening, eligibility assessment, and inclusion. Using the predefined search strategy, we retrieved 3,146 records from database searches. After removing 1,123 duplicates, 2,023 unique records remained for title and abstract screening. Of these, 1,872 records were excluded for not meeting the prespecified eligibility criteria. We then assessed 149 full-text articles for eligibility and excluded 132 with documented reasons (e.g., no extractable hazard ratio with 95% confidence interval, inappropriate comparator, overlapping cohorts without a unique outcome (20–22), or a non-MCI baseline). In total, 17 studies were included in the quantitative synthesis (meta-analysis).

Table 1 presents the description of the included studies. Overall, the evidence base comprised prospective cohort studies and analyses of large longitudinal datasets conducted across Europe (Spain, Italy, Belgium, Austria, the Netherlands, and the UK), North America (USA), and Asia (Japan and South Korea), as well as multi-cohort global consortia (COSMIC). Sample sizes ranged widely from small clinic-based cohorts (e.g., *n* = 61) to national administrative datasets (up to *n* = 336,313), with follow-up spanning approximately 6 months to 12 years. Three studies drawing on the National Alzheimer's Coordinating Center (NACC) dataset were included as separate entries because they reported different outcomes and analytic specifications (e.g., progression to AD vs. all-cause dementia, and unadjusted vs. adjusted models), as shown in Table 1.

**Table 1:** Description of the included studies.

Study	Study period	Country	Study name or clinic name	Total assessed	Follow up period	Progression	Effect of anxiety
Modrego and Ferrández (23)	June 1999 To June 2001	Spain	No Name	114	3 Years	AD	Unadjusted
Palmer et al. (24)	Not provided	Italy	Memory Clinics in Rome, Italy	131	Up To 4 Years	AD	Both
Rosenberg et al. (25)	Starting 2002	USA	Uniform Data Set Within NACC	1,821	1.5 Years	Dementia	Unadjusted
Van Der Mussele et al. (26)	Diagnosis since 2003	Belgium	Tertiary Care Memory Clinic	183	At Least 1 Year Follow Up Data	AD	Unadjusted
Forrester et al. (27)	September 2005 And August 2013	USA	No Name	540	2.00 (1.03) Years Mean Follow Up	Dementia	Multivariable
Kida et al. (28)	Since May 2001	Japan	Tone Project	276	Three Years	AD	Multivariable, two cohorts
Makizako et al. (29)	2011 and 2012	Japan	OSHPE	3,663	2 Years	Dementia	Unadjusted
Cova et al. (30)	January 2003 and May 2014	Italy	No Name	282	746.5 Person-Years Of Follow-Up	Dementia	Multivariable, two cohorts
Defrancesco et al. (31)	2005 to 2015	Austria	No Name	260	3.2 ± 2.2 Years	AD	Both
Ruthirakuhan et al. (32)	September 2005 and February 2018	USA	NACC	4,932	Not Provided	AD	Unadjusted
van De Beek et al. (33)	Not provided	Netherlands	Amsterdam Dementia Cohort	61	3.0 ± 2.0 Years.	Dementia	Unadjusted, two cohorts
Marquié et al. (34)	2016 - 2022	Spain	No Name	500	2.08 (1.2) Years	Dementia	Unadjusted
Qin et al. (35)	June 2005 -February 2021	China (USA data)	NACC	6,651	4 Years (Iqr 2, 6 Years)	Dementia	Multivariable
Willmott et al. (36)	1st January 2007 And 31st March 2019	UK	CRIS	2,250	At Least 6 Months	Dementia	Multivariable, two cohorts
Baik et al. (37)	2009 And 2015	South Korea	KNHIS	336,313	12 Years	AD	Both
Ding et al. (38)	Accessed In September 2024	USA	ADNI	397	3 Years	AD	Unadjusted, two cohorts
Oh et al. (39)	Varied	Global - 10 Population Cohorts - Six Continents	COSMIC	12,646	5.7 Years	Dementia	Multivariable, two cohorts

AD, Alzheimer's Disease; ADNI, Alzheimer's Disease Neuroimaging Initiative; COSMIC, Cohort Studies of Memory in an International Consortium; CRIS, Clinical Record Interactive Search; KNHIS, The Korean National Health Insurance Service; OSHPE, Obu Study of Health Promotion for the Elderly; NACC, The National Alzheimer's Coordinating Center; UK, United Kingdom; USA, United States of America.

### 3.2 Meta-analysis results

**Figure 2:**
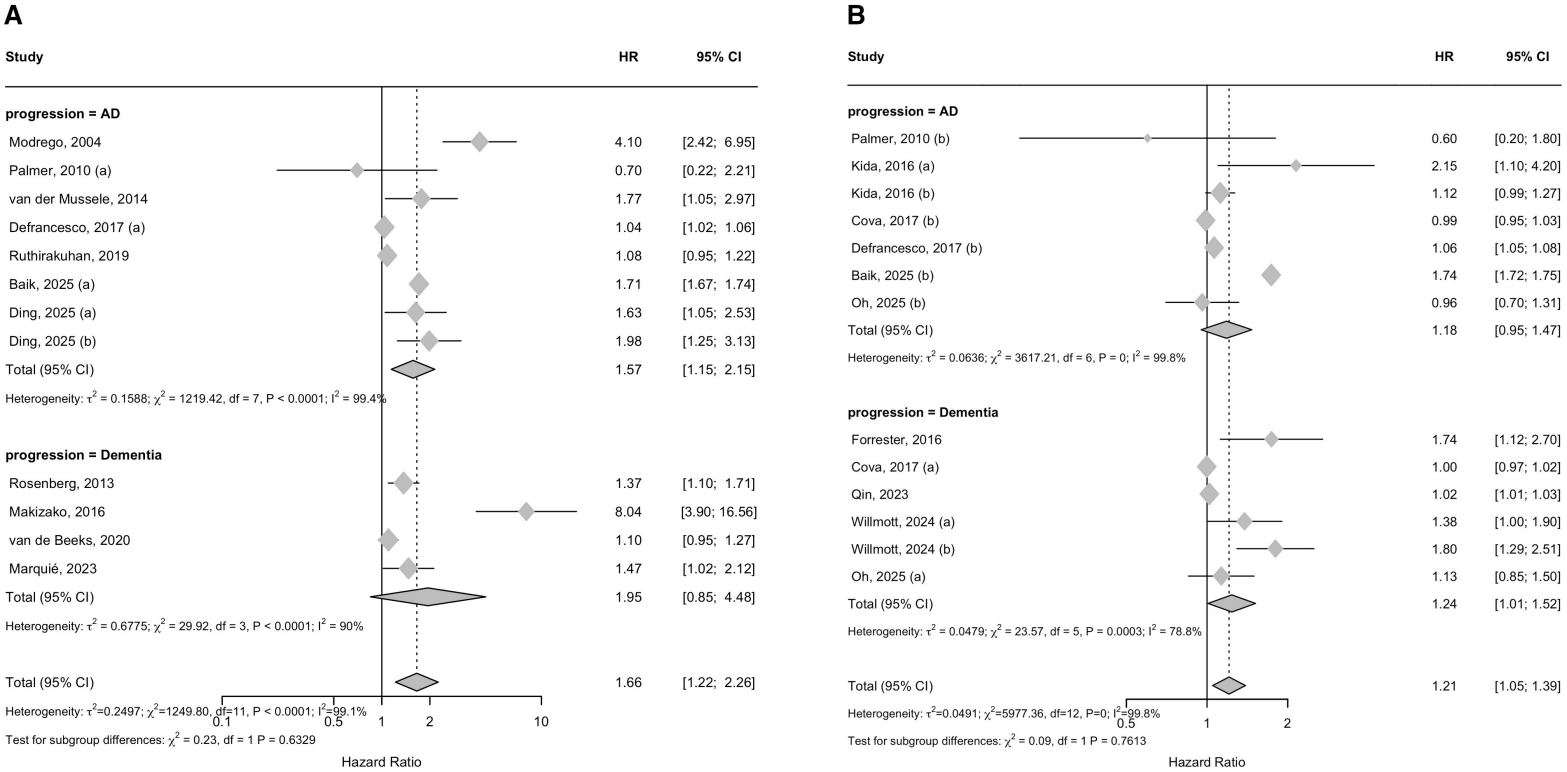
Forest plots of the association between baseline depression and risk of progression from mild cognitive impairment to Alzheimer's disease or all-cause dementia: **(A)** pooled unadjusted hazard ratios; **(B)** pooled adjusted hazard ratios.

Meta-analysis results are presented in Figure 2. Figure 2A summarizes pooled unadjusted HRs for progression from MCI to AD and to all-cause dementia. In the unadjusted analyses, baseline depression was associated with a higher risk of progression overall (HR 1.66, 95% CI 1.22–2.26), with high heterogeneity (*I*^2^ = 99.1%, *p* < 0.0001). The pooled association was statistically significant for progression to AD (HR 1.57, 95% CI 1.15–2.15), with high heterogeneity (*I*^2^ = 99.4%, *p* < 0.0001). However, the pooled estimate for progression to all-cause dementia was not statistically significant (HR 1.95, 95% CI 0.85–4.48) and showed substantial heterogeneity (*I*^2^= 90%, *p* < 0.0001).

In adjusted analyses (Figure 2B), baseline depression remained associated with increased progression risk overall (HR 1.21, 95% CI 1.05–1.39), with high heterogeneity (*I*^2^ = 99.8%, *p* < 0.001). The association was not statistically significant for progression to AD (HR 1.18, 95% CI 0.95–1.47), with high heterogeneity (*I*^2^ = 99.8%, *p* < 0.001). The pooled estimate for progression to all-cause dementia was statistically significant (HR 1.24, 95% CI 1.01–1.52), again with high heterogeneity (*I*^2^ = 78.8%, *p* = 0.0003). Notably, heterogeneity was extremely high across most pooled analyses, indicating substantial variability between studies and limiting the interpretability of pooled effect estimates.

### 3.3 Assessment of heterogeneity and publication bias assessment

**Figure 3:**
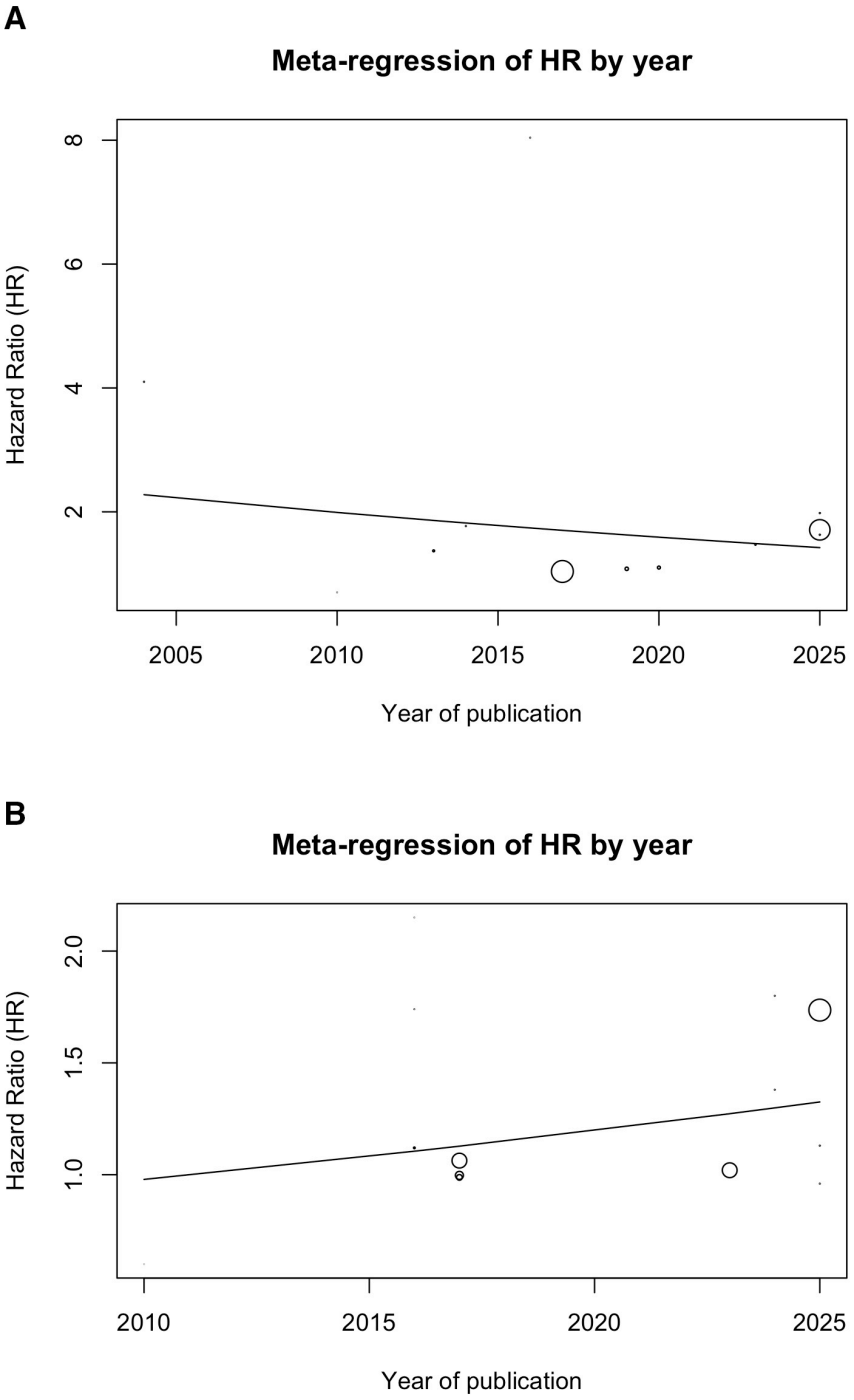
Meta-regression by publication year for: **(A)** pooled unadjusted hazard ratios; **(B)** pooled adjusted hazard ratios.

Meta-regression analyses did not indicate an association between publication year and effect size (Figures 3A, B). In the unadjusted model, year of publication was not a significant predictor of the pooled hazard ratio (*p* = 0.38). Similarly, in the adjusted model, no significant temporal trend was observed (*p* = 0.23). These findings suggest that publication year did not explain the substantial between-study heterogeneity observed, indicating that other methodological and clinical factors likely contribute to the variability in effect estimates.

**Figure 4:**
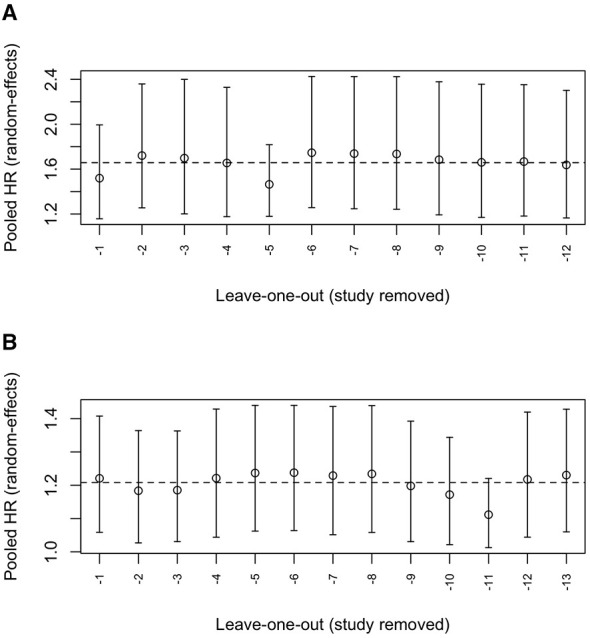
Leave-one-out analysis for: **(A)** pooled unadjusted hazard ratios; **(B)** pooled adjusted hazard ratios.

Leave-one-out sensitivity analyses are presented in Figure 4. In both the unadjusted (Figure 4A) and adjusted (Figure 4B) models, sequential removal of individual studies produced pooled hazard ratios that remained close to the overall random-effects estimate and within overlapping confidence intervals. No single study meaningfully altered the magnitude or direction of the association, indicating that the overall findings were robust and not driven by any individual study.

**Figure 5:**
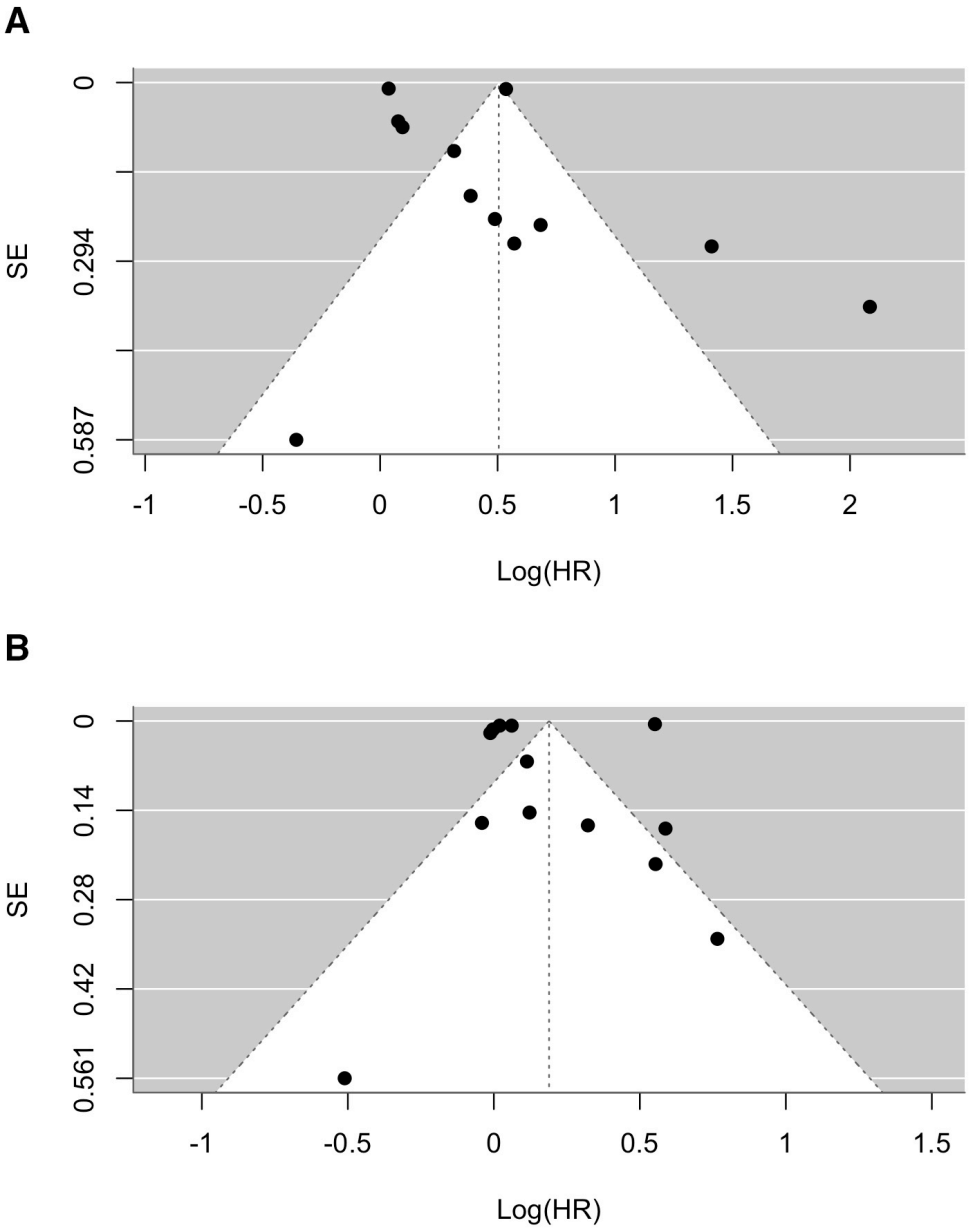
Funnel plot assessing small-study effects for: **(A)** pooled unadjusted hazard ratios; **(B)** pooled adjusted hazard ratios.

Visual inspection of the funnel plots (Figures 5A, B) did not provide strong evidence of small-study effects. In the unadjusted analysis, Egger's test was not significant (*p* = 0.0647), indicating at most weak evidence of small-study effects. Egger's regression test was not statistically significant in the adjusted analysis (*p* = 0.3828), suggesting no evidence of publication bias.

### 3.4 Risk of bias and certainty of evidence evaluation

Risk of bias assessments using the NOS are summarized in Table 2. Overall, study quality was predominantly moderate to high, with 9 studies rated as moderate quality and 8 studies rated as high quality. Across the NOS domains, most studies scored well on selection and outcome assessment, indicating generally appropriate cohort selection and outcome ascertainment. No study was rated as low quality.

**Table 2:** Risk of bias assessment of included cohort studies using the Newcastle–Ottawa Scale.

S. No	Study	Study name or clinic name	Selection (0–4^*^)	Compara bility (0–2^*^)	Outcome (0–3)^*^	Quality
			4 items	1 item	3 items	
1	Modrego and Ferrández (23)	No Name	^**^	^*^	^***^	Moderate
2	Palmer et al. (24)	Memory Clinics in Rome, Italy	^***^	^**^	^***^	High
3	Rosenberg et al. (25)	Uniform Data Set Within NACC	^****^	^**^	^***^	High
4	Van Der Mussele et al. (26)	Tertiary Care Memory Clinic	^**^	^*^	^***^	Moderate
5	Forrester et al. (27)	No Name	^***^	^*^	^**^	Moderate
6	Kida et al. (28)	Tone Project	^****^	^**^	^***^	High
7	Makizako et al. (29)	OSHPE	^***^	^*^	^***^	Moderate
8	Cova et al. (30)	No Name	^***^	^*^	^***^	Moderate
9	Defrancesco et al. (31)	No Name	^***^	^*^	^***^	Moderate
10	Ruthirakuhan et al. (32)	NACC	^****^	^**^	^***^	High
11	van de Beek et al. (33)	Amsterdam Dementia Cohort	^***^	^*^	^**^	Moderate
12	Marquié et al. (34)	No Name	^**^	^*^	^***^	Moderate
13	Qin et al. (35)	NACC	^****^	^**^	^***^	High
14	Willmott et al. (36)	CRIS	^****^	^**^	^***^	High
15	Baik et al. (37)	KNHIS	^**^	^*^	^***^	Moderate
16	Ding et al. (38)	ADNI	^****^	^**^	^***^	High
17	Oh et al. (39)	COSMIC	^****^	^**^	^***^	High

ADNI, Alzheimer's Disease Neuroimaging Initiative; COSMIC, Cohort Studies of Memory in an International Consortium; CRIS, Clinical Record Interactive Search; KNHIS, The Korean National Health Insurance Service; OSHPE, Obu Study of Health Promotion for the Elderly; NACC, The National Alzheimer's Coordinating Center. In the Newcastle-Ottawa Scale, the symbol “^*^” indicates one point awarded for meeting a quality criterion. A maximum of four stars can be assigned for Selection, two stars for Comparability, and three stars for Outcome assessment. Studies with 7-9 stars were considered high quality, 4-6 stars moderate quality, and ≤ 3 stars low quality.

Outcome-level GRADE assessment results are summarized in Table 3. The certainty of evidence for both pooled estimates was rated very low, reflecting limited confidence in the magnitude of effect. For the unadjusted pooled analysis (12 studies; 348,375 participants; pooled HR 1.66, 95% CI 1.22–2.26), evidence was downgraded primarily for serious inconsistency, indicating substantial between-study variability, and for suspected publication bias. Risk of bias was not judged serious, and there were no major concerns regarding indirectness or imprecision. Similarly, for the adjusted pooled analysis (13 studies; 359,601 participants; pooled HR 1.21, 95% CI 1.05–1.39), the certainty of evidence was also rated very low, driven mainly by serious inconsistency across studies. Indirectness and imprecision were not considered serious, publication bias was not detected, and no factors justified upgrading. Given the very low certainty of evidence, these findings should be interpreted—and any clinical or policy actions based on them considered—with caution.

**Table 3:** Outcome-level GRADE certainty of evidence assessment.

Outcome	No. studies	Participants	Pooled HR (95% CI)	Certainty (GRADE)	Risk of bias	Inconsistency	Indirectness	Imprecision	Publicationbias	Other considerations (upgrade)
Unadjusted pooled HR	12	348,375	1.66 (1.22–2.26)	Very low	Not serious	Serious	Not serious	Not serious	Suspected	None
Adjusted pooled HR	13	359,601	1.21 (1.05–1.39)	Very low	Not serious	Serious	Not serious	Not serious	None	None

HR, Hazard ratio.

## 4 Discussion

This systematic review and meta-analysis synthesized evidence from 17 longitudinal cohort studies across diverse populations and settings. The pooled results suggest that baseline depression in individuals with MCI was associated with an increased risk of progression to dementia. In unadjusted analyses, depression significantly predicted conversion to AD (HR 1.57, 95% CI 1.15–2.15), while the association with all-cause dementia was not statistically significant. In adjusted models, depression remained a significant predictor for all-cause dementia, while the association with AD was attenuated and no longer statistically significant. This attenuation suggests that part of the observed association in unadjusted analyses may be explained by confounding clinical and demographic factors. Taken together, these results suggest that depression may be linked to faster clinical deterioration in MCI; however, the extremely high heterogeneity across studies substantially limits confidence in the pooled estimates and their generalizability.

The observed association is likely not explained by a single mechanism. One possibility is that depression in later life acts as a prodromal signal of underlying neurodegenerative processes rather than an independent causal factor (40, 41). In this context, depressive symptoms may emerge alongside early cognitive and behavioral changes, making it difficult to separate psychiatric manifestations from the earliest stages of dementia.

At the same time, depression may also contribute to disease progression through pathways that reduce cognitive reserve or increase biological vulnerability. Evidence suggests that chronic stress and inflammation compromise vascular and brain function, with pro-inflammatory cytokines and microglial activation driving neurodegenerative changes (11). Reduced cognitive reserve has been shown to heighten vulnerability to dementia in individuals with depression, indicating that lower resilience may exacerbate disease progression (42). Dysregulation of the hypothalamic–pituitary–adrenal axis and glucocorticoid excess further weaken neural resilience and contribute to hippocampal atrophy (43). Sleep disturbance, commonly observed in depression, has been linked to impaired clearance of neurotoxic proteins such as β-amyloid, thereby accelerating decline (44). Finally, diminished social and physical engagement reduces protective factors such as neuroplasticity and cognitive reserve, increasing susceptibility to dementia (45). A clinically important implication is that depression in MCI should not be viewed as a purely “reactive” symptom; it may reflect both treatable psychopathology and evolving brain pathology.

The findings should also be interpreted in the context of symptom timing and chronicity. Prior studies suggest that late-onset or new-onset depression may carry a different prognostic meaning than recurrent or remote depression, and persistent symptoms may indicate greater vulnerability than episodic symptoms (46–48). This may partly account for variability across studies, as many cohorts classified depression as a binary exposure without distinguishing onset, duration, or severity. In addition, co-occurring neuropsychiatric symptoms, such as anxiety, apathy, and chronic stress, may influence progression and may confound or modify the observed associations (49, 50).

Our review also highlights the role of symptom clustering, rather than depression alone. Anxiety, apathy, sleep disruption, and chronic stress frequently co-occur with depression and may influence progression through overlapping but non-identical pathways (39, 49, 51–53). In practice, patients with MCI often present with mixed neuropsychiatric profiles, and these combined features may carry greater prognostic value than depression alone. This may partly explain why adjusted associations differ across studies: covariate sets vary substantially, and some studies adjust for neuropsychiatric comorbidity while others do not. A broader neuropsychiatric-risk model may therefore be more informative than evaluating depression in isolation.

This review adds to the literature by quantifying the prognostic association between depression and dementia outcomes in MCI using longitudinal evidence. However, heterogeneity was substantial across pooled analyses. Likely sources include differences in MCI diagnostic criteria, depression measurement (self-report scales vs. clinical diagnosis), follow-up duration, outcome definitions, and covariate adjustment. Depression ascertainment is particularly important: self-report measures may capture transient distress or subthreshold symptoms, while clinical diagnosis may better identify persistent depressive disorders (47, 54). These differences may contribute to inconsistency in pooled estimates. Although meta-regression by publication year did not explain heterogeneity, the ability to formally explore other sources (e.g., depression assessment method or follow-up duration) was limited by inconsistent reporting and the relatively small number of studies per subgroup.

The findings have implications for future research. Prospective studies should use more standardized and transparent definitions of depression, including symptom severity, onset, and persistence. Repeated measurement of depressive symptoms over time would improve understanding of whether persistent, remitting, or late-onset depression is most strongly associated with progression. Future studies should also report dementia subtypes consistently and use comparable adjustment sets. Where possible, integration of neuropsychiatric data with biomarkers and neuroimaging may help clarify whether depression acts primarily as a modifiable risk factor, a prodromal marker, or both.

Several limitations of this review should be considered. First, the overall certainty of evidence was very low, largely due to between-study heterogeneity and methodological limitations in the primary studies. Second, residual confounding remains likely because adjustment was inconsistent and not all studies accounted for comorbid illness, medication exposure, social isolation, or lifestyle factors. Third, observational designs preclude firm causal conclusions; therefore, the results should be interpreted as prognostic rather than causal evidence. Fourth, publication bias cannot be fully excluded, even if formal assessments did not strongly suggest small-study effects. Additionally, variability in covariate adjustment across studies limits the comparability of adjusted estimates and complicates interpretation of pooled results.

## 5 Practical implications and conclusion

The clinical message is that depression in patients with MCI should be treated as a high-priority prognostic signal. Even when causality is uncertain, depressive symptoms identify a subgroup that may benefit from closer follow-up, repeated cognitive assessment, and more comprehensive diagnostic workup. In clinical practice, this means that detection of depression in MCI should trigger not only psychiatric management but also structured monitoring for cognitive progression. Distinguishing long-standing depression from late-onset or worsening depression may be particularly important, as these patterns may reflect different risk profiles.

From a public health perspective, the results suggest integrating mental health screening into memory care and dementia prevention pathways. Depression assessment is low-cost and scalable, and it may improve risk stratification when combined with cognitive testing and clinical history. Programs targeting older adults with MCI could benefit from a dual approach: active treatment of depressive symptoms to improve quality of life and functioning, alongside surveillance for early neurodegenerative progression.

In conclusion, depression may represent a potential prognostic factor for progression in MCI; however, given the substantial heterogeneity and very low certainty of evidence, these findings should be interpreted cautiously. Higher-quality longitudinal studies are needed to clarify mechanisms and identify which depressive phenotypes are most strongly associated with dementia progression.

## Data Availability

The original contributions presented in the study are included in the article/supplementary material, further inquiries can be directed to the corresponding author.
